# The consideration of surgery on primary lesion of advanced non-small cell lung cancer

**DOI:** 10.1186/s12890-023-02411-w

**Published:** 2023-04-14

**Authors:** Jianghao Ren, Jiangbin Ren, Kan Wang, Qiang Tan

**Affiliations:** 1 Department of Thoracic Surgery, Shanghai Chest Hospital, Shanghai Jiaotong University, 241 Huaihai Rd, Shanghai, 200030 China; 2 Huai’an First People’s Hospital, Nanjing Medical University, Huai’an, Jiangsu China; 3 The 4Th Affiliated Hospital of Harbin Medical University, Harbin, China

**Keywords:** Lung cancer, Treatments, Palliative, Surgery

## Abstract

**Background:**

Numerous reports have shown that medical treatment confers excellent survival benefits to patients with advanced stage IV non-small cell lung cancer (NSCLC). However, the implications of surgery for primary lesions as palliative treatment remain inconclusive.

**Methods:**

We retrospectively extracted clinical data from the Surveillance, Epidemiology, and End Results Program (SEER) database and selected patients with stage IV NSCLC. Patients were classified into non-surgery and surgery groups, and propensity score matching (PSM) analysis was performed to balance the baseline information. Patients in the surgery group, whose overall survival (OS) was longer than the median survival time of those in the non-surgery group, were deemed to benefit from surgery. We evaluated the efficacy of three surgical techniques, namely, local destruction, sub-lobectomy, and lobectomy, on the primary site in the beneficial population.

**Results:**

The results of Cox regression analyses revealed that surgery was an independent risk factor for both OS (hazard ratio [HR]: 0.441; confidence interval [CI]: 0.426–0.456; *P* < 0.001) and cancer-specific survival (CSS) (HR: 0397; CI: 0.380–0.414; *P* < 0.001). Notably, patients who underwent surgery had a better prognosis than those who did not (OS: *P* < 0.001; CSS: *P* < 0.001). Moreover, local destruction and sub-lobectomy significantly compromised survival compared to lobectomy in the beneficial group (*P* < 0.001). After PSM, patients with stage IV disease who underwent lobectomy needed routine mediastinal lymph node clearing (OS: *P* = 0.0038; CSS: *P* = 0.039).

**Conclusion:**

Based on these findings, we recommend that patients with stage IV NSCLC undergo palliative surgery for the primary site and that lobectomy plus lymph node resection should be conventionally performed on those who can tolerate the surgery.

## Introduction

According to the Global Cancer Statistics of 2020, approximately 19.3 million new cases and 10 million cancer-related deaths occurred in 2020 alone, with lung cancer being classified as the most lethal cancer [1]. Current advancements in diagnostics and medical technology have contributed to significant improvement in the survival of patients with non-small cell lung cancer (NSCLC), which accounts for 80% of all lung cancer cases. In the United States, the 5-year survival rate of patients with NSCLC has improved from 16.4% to 25.1% from 1975 to 2015. However, almost 55% of these patients eventually develop advanced lung cancers [2, 3]. Although immune therapy and targeted drugs have significantly improved patient prognosis, the future of advanced tumour treatment remains unknown, necessitating further research. Notably, surgery is generally not recommended for patients with advanced NSCLC, especially for those with distant progression. Nevertheless, some scholars have suggested that surgical operations should be expanded to include patients with stage IV NSCLC, especially those with oligometastatic tumours [4–7]. However, the benefit of surgery in patients with advanced NSCLC is inconclusive, and the most optimal surgical technique among local destruction, ablation therapy, sub-lobectomy, and lobectomy, remains unknown [8]. In the present study, we analysed the clinical information of patients with stage IV NSCLC from the Surveillance, Epidemiology, and End Results Program (SEER) database and investigated the implications of surgery on survival outcomes.

## Methods

### Patient selection

Clinical data for patients diagnosed with NSCLC between 2004–2016 (C34.0–C34.9) were extracted from the SEER database. A total of 122,650 patients with stage IV NSCLC were retrospectively selected, and the surgical codes for the primary site were set as 00 (non-surgery); 12, 13, and 15 (local destruction: ablation); 21 and 22 (sub-lobectomy); and 30 and 33 (lobectomy with or without mediastinal lymph node clearing). Pathologies were defined as large cell carcinoma, adenocarcinoma, squamous cell carcinoma, adenosquamous carcinoma, and neuroendocrine carcinoma. Patients with small cell lung cancer, as well as those with unknown TNM stage and unknown survival status, including cancer-specific survival (CSS), were excluded from the list.

### Study design

**Fig. 1:**
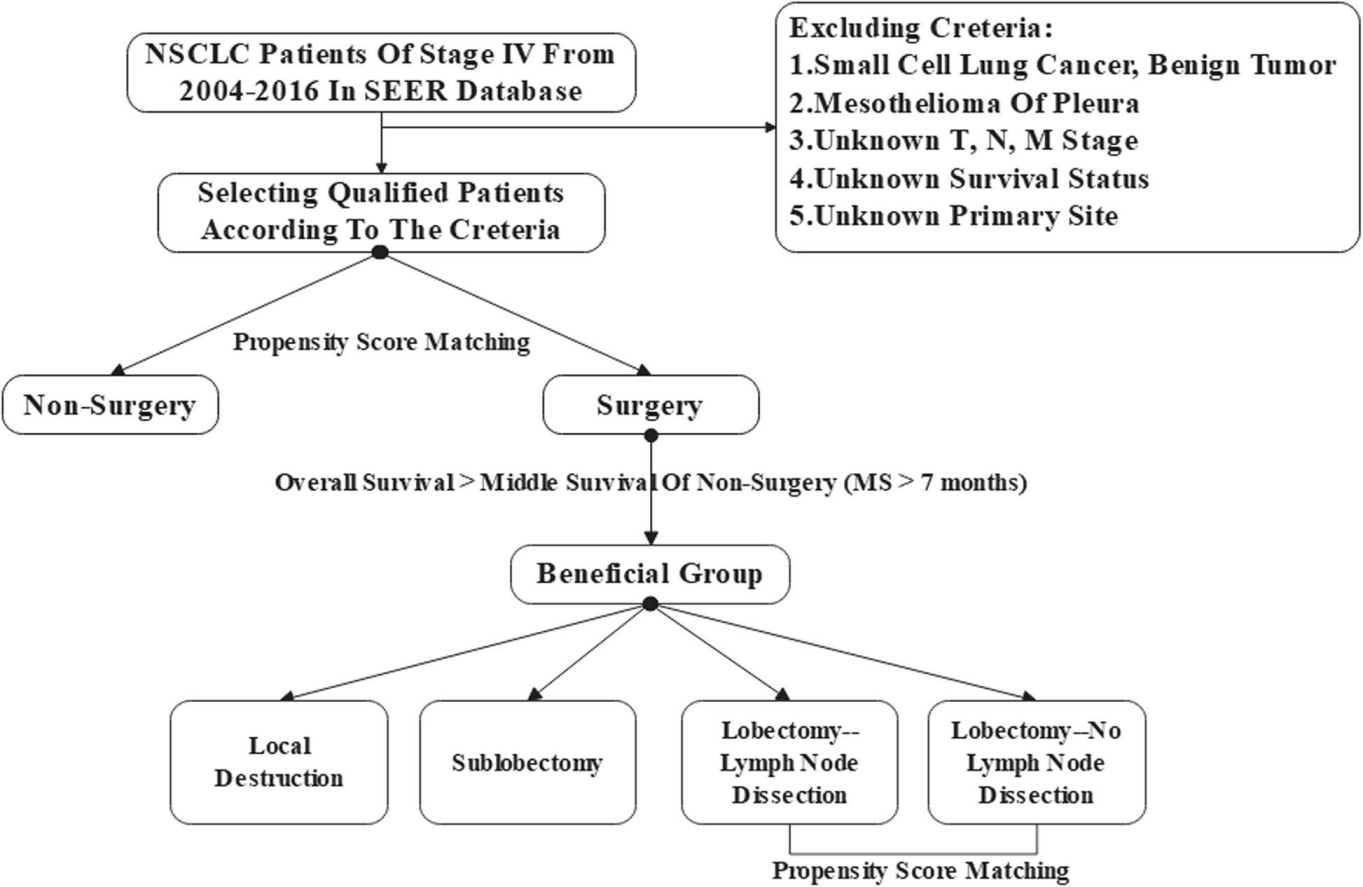
Creteria of patients selection and the study design

Eligible patients were divided into the non-surgery group (code 00) and surgery group (codes 12, 13, 15, 21, 22, 30, and 33), based on the operation on the primary site. Further, we compared the long-term outcomes between the groups after balancing their baseline information. Notably, we classified patients whose median survival times were greater than those in the non-surgical group into the benefit group. We compared different outcomes among the three surgical methods: local destruction, lobectomy, and sub-lobectomy, including wedge resection and segmental resection. Furthermore, we investigated the significance of lymph node dissection in the lobectomy group after balancing the baseline characteristics. The TNM stage in this study was reclassified according to the American Joint Committee on Cancer (AJCC), 8^th^ version, and the outcomes were overall survival (OS) and CSS (Fig. 1).

### Statistical analysis

Statistical analyses were performed using SAS version 9.4 and packages implemented in R software version 4.0.3. Continuous variables are expressed as means ± standard deviations. Differences in continuous variables between the two groups were compared using independent two-sample *t*-test, whereas those for categorical variables were analysed using Fisher’s exact or chi-square tests. Baseline characteristics between the surgery and non-surgery groups were balanced using propensity score matching (PSM; Caliper = 0.001). Further, we applied the product-limit algorithm implemented using the Kaplan–Meier method and the log-rank test to evaluate OS and CSS. Thereafter, we performed Cox regression analyses based on univariate and multivariate methods to determine the significance of surgery in stage IV NSCLC (Method = “enter”). We also applied the nearest propensity score on the logit scale to a 1:1 match (Caliper = 0.01) for the lymph node dissection and non-dissection groups to evaluate the implication of lymph node resection in the lobectomy group. Additionally, we manually converted multiple categorical variables to dummy variables for regression analysis. Statistical significance was set at *P* ≤ 0.05.

## Results

### Baseline characteristics of patients with stage IV NSCLC

A summary of the baseline characteristics of the two groups is presented in Table 1. A total of 122,650 patients were included, of whom 3.70% underwent surgery. Before PSM, we collected clinical information regarding age, race, sex, laterality, position, T stage, N stage, M stage, clinical stage, grade, histology, radiation, chemotherapy, and distant progression. Almost all variables were unbalanced between the surgical and non-surgical groups. Notably, the lesions were more common in the upper lobes. Additionally, adenocarcinoma accounted for the majority of pathological types, with most of the patients having grade III and stage IVa. After PSM, we found a total of 4,232 patients in the two matched groups, and all baseline variables were 1:1 matched and finally balanced (Table 1).

**Table 1:** Baseline characteristics of patients with advanced NSCLC before PSM and after PSM

	Before PSM				After PSM			
Variable	Summarize (*n* = 122,650)	Non-surgery (*n* = 118,115), n(%)	Surgery (*n* = 4535), n(%)	*P* Value	Summarize (*n* = 8486)	Non-surgery (*n* = 4243), n(%)	Surgery (*n* = 4243), n(%)	*P* Value
Age (y)	68.21 ± 11.18	68.29 ± 11.19	66.13 ± 10.69	< 0.001	66. ± 11.09	66.16 ± 11.53	66.25 ± 10.67	0.732
Race				< 0.001				0.439
White	97,538	93,724 (79.3)	3814 (84.1)		7168	3599 (84.8)	3569 (84.1)	
Black	15,814	15,354 (13.0)	460 (10.1)		835	400 (9.4)	435 (10.3)	
Others	9289	9037 (7.7)	261 (5.8)		483	244 (5.8)	239 (5.6)	
Gender				< 0.001				0.422
Male	68,586	66,246 (56.1)	2340 (51.6)		4365	2164 (51.0)	2201 (51.9)	
Female	54,064	51,869 (43.9)	2195 (48.4)		4121	2079 (49.0)	2042 (48.1)	
Laterality				0.570				0.861
Left	50,682	48,841 (41.4)	1841 (40.6)		3468	1737 (40.9)	1731 (40.8)	
Right	71,507	68,829 (58.3)	2678 (59.1)		4985	2488 (58.6)	2497 (58.8)	
Bilateral	461	445 (0.3)	16 (0.3)		33	18 (0.5)	15 (0.4)	
Surgical Position				< 0.001				0.990
Upper Lobe	73,558	70,915 (60.0)	2643 (58.3)		4955	2482 (58.5)	2473 (58.3)	
Middle Lobe	5788	5510 (4.7)	278 (6.1)		515	259 (6.1)	256 (6.0)	
Lower Lobe	35,599	34,149 (28.9)	1450 (32.0)		2703	1350 (31.8)	1353 (31.9)	
Main Bronchus	6246	6159 (5.2)	87 (1.9)		168	82 (1.9)	86 (2.0)	
Overlapping Lesion	1459	1382 (1.2)	77 (1.7)		145	70 (1.7)	75 (1.8)	
T Stage				< 0.001				0.250
T1	17,265	16,368 (13.9)	897 (19.8)		1795	929 (21.9)	866 (20.4)	
T2	24,321	23,181 (19.6)	1140 (25.1)		2079	1035 (24.4)	1044 (24.6)	
T3	24,798	23,836 (20.2)	962 (21.2)		1758	887 (20.9)	871 (20.5)	
T4	56,266	54,730 (46.3)	1536 (33.9)		2854	1392 (32.8)	1462 (34.5)	
N Stage				< 0.001				0.773
N0	31,686	29,283 (24.8)	2403 (53.0)		4430	2225 (52.4)	2205 (52.0)	
N1	10,502	9843 (8.3)	659 (14.5)		1225	610 (14.4)	615 (14.5)	
N2	58,038	56,770 (48.1)	1268 (28.0)		2409	1189 (28.0)	1220 (28.8)	
N3	22,424	22,219 (18.8)	205 (4.5)		422	219 (5.2)	203 (4.7)	
M Stage				< 0.001				0.119
M1a	44,654	42,229 (35.8)	2425 (53.5)		4454	2212 (52.1)	2242 (52.8)	
M1b	28,984	28,286 (23.9)	698 (15.4)		1253	627 (14.8)	626 (14.8)	
M1c	11,271	11,190 (9.5)	81 (1.8)		193	113 (2.7)	80 (1.9)	
M1-Nos	37,741	36,410 (30.8)	1331 (29.3)		2586	1291 (30.4)	1295 (30.5)	
Clinical Stage				< 0.001				0.055
IVa	73,638	70,515 (59.7)	3123 (68.9)		5707	2839 (66.9)	2868 (67.6)	
IVb	11,271	11,190 (9.5)	81 (1.8)		193	113 (2.7)	80 (1.9)	
IV-Nos	37,741	36,410 (30.8)	1331 (29.3)		2586	1291 (30.4)	1295 (30.5)	
Grade				< 0.001				0.594
I	2535	2269 (1.9)	266 (5.9)		517	268 (6.3)	249 (5.9)	
II	14,481	13,049 (11.0)	1432 (31.6)		2635	1326 (31.3)	1309 (30.9)	
III	35,092	33,020 (28.0)	2072 (45.7)		3820	1878 (44.3)	1942 (45.8)	
IV	2220	2049 (1.7)	171 (3.8)		316	156 (3.7)	160 (3.8)	
Unknown	68,322	67,728 (57.3)	594 (13.1)		1198	615 (14.5)	583 (13.7)	
Histology				< 0.001				0.873
Large Cell	4023	3787 (3.2)	236 (5.2)		448	223 (5.3)	225 (5.3)	
Adenocarcinoma	64,178	61,412 (52.0)	2766 (61.0)		2593	2593 (61.1)	2548 (60.1)	
Squamous Cell	27,116	26,110 (22.1)	1006 (22.2)		927	927 (21.8)	970 (22.9)	
Adenosquamous	1606	1458 (1.2)	148 (3.3)		129	129 (3.0)	122 (2.9)	
Neuroendocrine	2835	2760 (2.3)	75 (1.6)		69	69 (1.6)	75 (1.8)	
NSCLC-Nos	22,892	22,588 (19.1)	304 (6.7)		302	302 (7.1)	303 (7.1)	
Radiation				< 0.001				0.720
No/Unknown	64,255	61,409 (52.0)	2846 (62.8)		5302	2659 (62.7)	2643 (62.3)	
Yes	58,395	56,706 (48.0)	1689 (37.2)		3184	1584 (37.3)	1600 (37.7)	
Chemotherapy				0.613				0.617
No/Unknown	56,993	54,869 (46.5)	2124 (46.8)		4021	2022 (47.7)	1999 (47.1)	
Yes	65,657	63,246 (53.5)	2411 (53.2)		4465	2221 (52.3)	2244 (52.9)	
Distant Progression								
Bone	25,629	25,318	311	< 0.001	752	466	286	
Brain	17,707	17,240	467	< 0.001	734	309	425	
Liver	11,731	11,620	111	< 0.001	255	151	104	
Lung	20,132	19,634	498	< 0.001	1133	695	438	

### Efficacy of surgery on OS and CSS

The variables with significant differences (*P* < 0.05) in the univariate analysis were selected for the multivariate Cox analysis (Table 2). Results from the Cox analysis indicated that surgical operation was a significant independent risk factor for both OS (hazard ratio [HR]: 0.441; confidence interval [CI]: 0.426–0.456; *P* < 0.001) and CSS (HR: 0.397; CI: 0.380–0.414; *P* < 0.001). Additionally, T, N, and M stages, as well as grade (*P* < 0.001), significantly correlated with the prognosis of patients with advanced NSCLC after surgery.

**Table 2:** Univariate and multivariate analyses of prognostic factors of OS and CSS

Variable	Overall Survival				Cancer-Specific Survival			
	Univariate Analyses		Multivariate analyses		Univariate Analyses		Multivariate analyses	
	HR(95%CI)	*p* Value	HR(95%CI)	*p* Value	HR(95%CI)	*p* Value	HR(95%CI)	*p* Value
Age at diagnosis	1.014(1.013–1.014)	< 0.001	1.008(1.007–1.009)	< 0.001	1.004(1.003–1.004)	< 0.001	0.999(0.999–1.000)	0.007
Race		<0.001		<0.001		<0.001		<0.001
White	Reference		Reference		Reference		Reference	
Black	0.992(0.975–1.009)	0.348	0.974(0.957–0.991)	0.003	1.031(1.011–1.052)	0.002	0.977(0.958–0.997)	0.024
Others	0.739(0.722–0.756)	< 0.001	0.749(0.732–0.767)	< 0.001	0.825(0.804–0.846)	< 0.001	0.821(0.800–0.842)	< 0.001
Gender								
Male	Reference		Reference		Reference		Reference	
Female	0.830(0.820–0.840)	< 0.001	0.854(0.844–0.864)	< 0.001	0.837(0.826–0.848)	< 0.001	0.866(0.854–0.878)	< 0.001
Laterality		0.201				<0.001		0.002
Left	Reference				Reference		Reference	
Right	1.009(0.997–1.021)	0.125			1.026(1.013–1.040)	< 0.001	1.021(1.007–1.035)	0.003
Bilateral	0.961(0.873–1.058)	0.417			0.913(0.815–1.024)	0.119	0.909(0.811–1.019)	0.101
Lesion Position		<0.001		<0.001		<0.001		<0.001
Upper Lobe	Reference		Reference		Reference		Reference	
Middle Lobe	0.945(0.919–0.972)	< 0.001	0.969(0.943–0.997)	0.029	0.922(0.893–0.953)	< 0.001	0.940(0.910–0.972)	< 0.001
Lower Lobe	1.007(0.994–1.020)	0.321	1.018(1.004–1.031)	0.009	0.988(0.973–1.003)	0.115	1.018(1.002–1.033)	0.023
Main Bronchus	1.219(1.188–1.252)	< 0.001	1.169(1.139–1.201)	< 0.001	1.268(1.230–1.306)	< 0.001	1.191(1.156–1.227)	< 0.001
Overlapping lesion	1.148(1.088–1.210)	< 0.001	1.121(1.063–1.183)	< 0.001	1.185(1.116–1.259)	< 0.001	1.149(1.081–1.220)	< 0.001
Surgery	0.415(0.401–0.429)	< 0.001	0.441(0.426–0.456)	< 0.001	0.359(0.345–0.375)	< 0.001	0.397(0.380–0.414)	< 0.001
T Stage		<0.001		<0.001		<0.001		<0.001
T1	Reference		Reference		Reference		Reference	
T2	1.150(1.126–1.173)	< 0.001	1.164(1.141–1.188)	< 0.001	1.187(1.159–1.216)	< 0.001	1.202(1.173–1.231)	< 0.001
T3	1.223(1.198–1.248)	< 0.001	1.236(1.211–1.262)	< 0.001	1.266(1.236–1.296)	< 0.001	1.274(1.244–1.304)	< 0.001
T4	1.374(1.350–1.399)	< 0.001	1.444(1.418–1.471)	< 0.001	1.453(1.423-.1484)	< 0.001	1.536(1.503–1.569)	< 0.001
N Stage		<0.001		<0.001		<0.001		<0.001
N0	Reference		Reference		Reference		Reference	
N1	1.115(1.089–1.140)	< 0.001	1.172(1.146–1.200)	< 0.001	1.189(1.157–1.221)	< 0.001	1.214(1.182–1.247)	< 0.001
N2	1.278(1.260–1.296)	< 0.001	1.308(1.289–1.327)	< 0.001	1.401(1.377–1.424)	< 0.001	1.376(1.353–1.400)	< 0.001
N3	1.226(1.204–1.248)	< 0.001	1.340(1.316–1.365)	< 0.001	1.385(1.357–1.414)	< 0.001	1.432(1.402–1.463)	< 0.001
M Stage		<0.001				<0.001		
M1a	Reference				Reference			
M1b	1.172(1.154–1.190)	< 0.001			1.261(1.239–1.284)	< 0.001		
M1c	1.496(1.465–1.529)	< 0.001			1.684(1.643–1.725)	< 0.001		
M1-Nos	1.239(1.221–1.256)	< 0.001			1.367(1.344–1.389)	< 0.001		
Clinical Stage		<0.001		<0.001		<0.001		<0.001
IVa	Reference		Reference		Reference		Reference	
IVb	1.409(1.380–1.438)	< 0.001	1.827(1.787–1.869)	< 0.001	1.538(1.503–1.574)	< 0.001	1.928(1.880–1,978)	< 0.001
IV-Nos	1.168(1.153–1.183)	< 0.001	1.411(1.390–1.432)	< 0.001	1.250(1.232–1.269)	< 0.001	1.509(1.483–1.536)	< 0.001
Grade		<0.001		<0.001		<0.001		<0.001
I	Reference		Reference		Reference		Reference	
II	1.246(1.191–1.303)	< 0.001	1.196(1.142–1.250)	< 0.001	1.318(1.248–1.391)	< 0.001	1.243(1.177–1.312)	< 0.001
III	1,592(1.525–1.662)	< 0.001	1.439(1.378–1.503)	< 0.001	1.742(1.654–1.835)	< 0.001	1.503(1.426–1.583)	< 0.001
IV	1.721(1.621–1.826)	< 0.001	1.491(1.402–1.585)	< 0.001	1.908(1.779–2.047)	< 0.001	1.551(1.443–1.667)	< 0.001
Unknown	1.582(1.516–1.651)	< 0.001	1.385(1.327–1.446)	< 0.001	1.735(1.649–1.826)	< 0.001	1.452(1.379–1.529)	< 0.001
Histology		<0.001		<0.001		<0.001		<0.001
Large Cell	Reference		Reference		Reference		Reference	
Adenocarcinoma	0.772(0.747–0.797)	< 0.001	0.848(0.819–0.877)	< 0.001	0.754(0.727–0.783)	< 0.001	0.835(0.803–0.868)	< 0.001
Squamous Cell	0.950(0.919–0.983)	0.003	0.939(0.906–0.973)	< 0.001	0.882(0.848–0.916)	< 0.001	0.908(0.872–0.946)	< 0.001
Adenosquamous	0.907(0.854–0.962)	0.001	0.968(0.911–1.028)	0.284	0.872(0.814–0.933)	< 0.001	0.946(0.882–1.014)	0.116
Neuroendocrine	0.946(0.901–0.994)	0.028	0.928(0.883–0.976)	0.003	0.932(0.881–0.986)	0.014	0.922(0.871–0.976)	0.005
NSCLC-Nos	0.995(0.961–1.029)	0.751	0.970(0.936–1.005)	0.088	0.987(0.949–1.026)	0.500	0.969(0.931–1.009)	0.128
Radiation	0.907(0.896–0.917)	< 0.001	0.894(0.883–0.905)	< 0.001	0.974(0.961–0.987)	< 0.001	0.921(0.908–0.934)	< 0.001
Chemotherapy	0.463(0.458–0.469)	< 0.001	0.437(0.431–0.442)	< 0.001	0.493(0.487–0.500)	< 0.001	0.434(0.428–0.441)	< 0.001

### Efficacy of surgery on the prognosis of patients with stage IV NSCLC

**Fig. 2:**
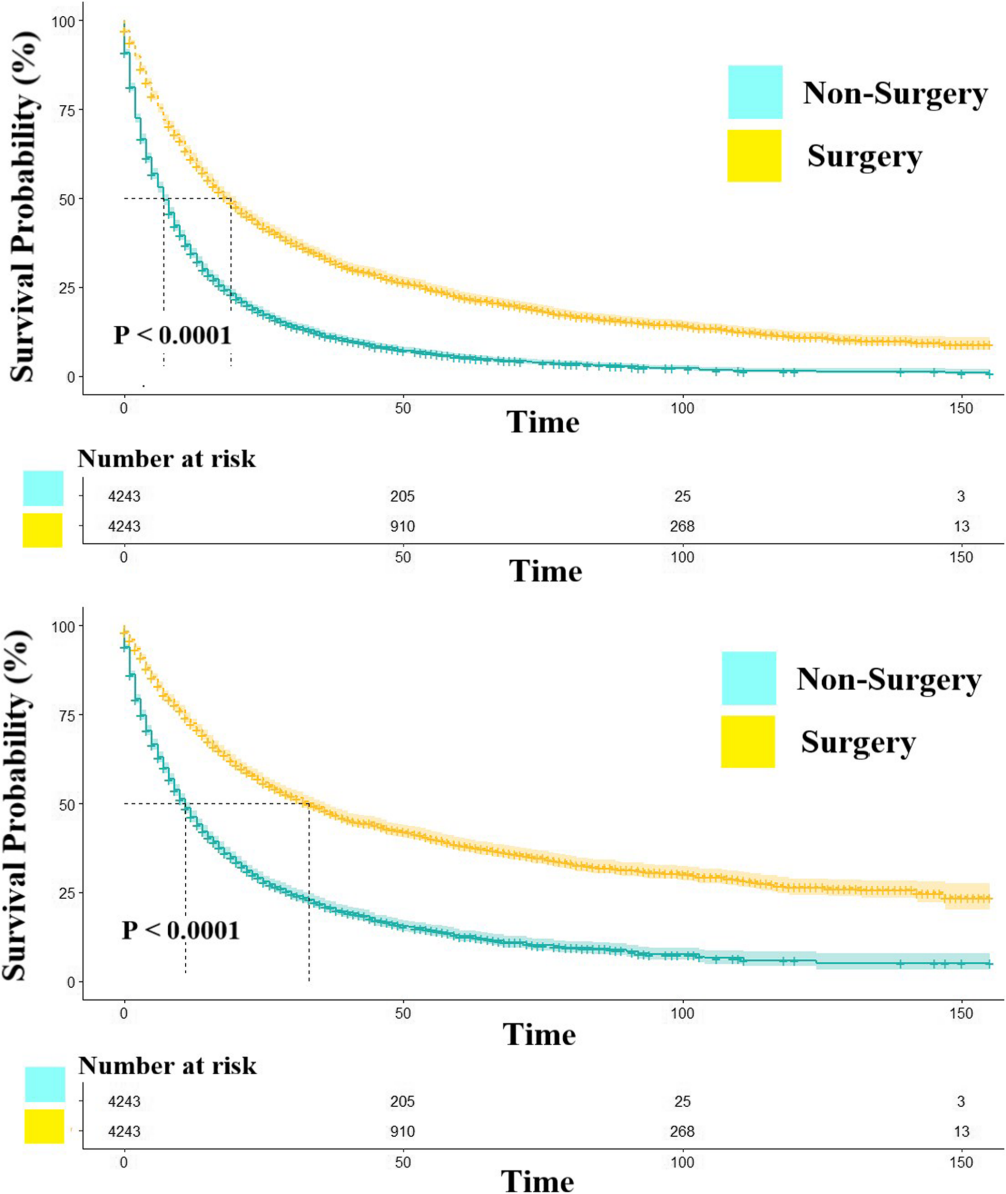
The OS and CSS of non-surgery and surgery groups after PSM. NOTES:Upper: Overall survival; Lower: Cancer-specific survival

We applied the log-rank test to compare the survival rates between patients in the non-surgery and surgery groups in the matched population after PSM and then generated Kaplan–Meier curves to investigate patient survival (Fig. 2). The results revealed that patients in the surgery group had a significantly better prognosis than those in the non-surgery group, regardless of OS or CSS (*P* < 0.001). The median survival (MS) times for OS were 7 and 19 months in the non-surgery and surgery groups, respectively. Regarding CSS, we found an MS of 11 and 33 months for the non-surgery and surgery groups, respectively. Results from the long-term follow-up revealed 1-, 5-, and 10-year OS rates of 34.5%, 5.4%, and 1.3%, respectively, for the non-surgery group, while those of the palliative surgery group were 61.2%, 22.4%, and 11.2%, respectively. Based on these survival rates, we stratified patients who had OS rates greater than the MS times in the non-surgery group into two categories, namely beneficial and non-beneficial groups (*P* < 0.001).

### Effect of surgical methods on the beneficial group after PSM

**Fig. 3:**
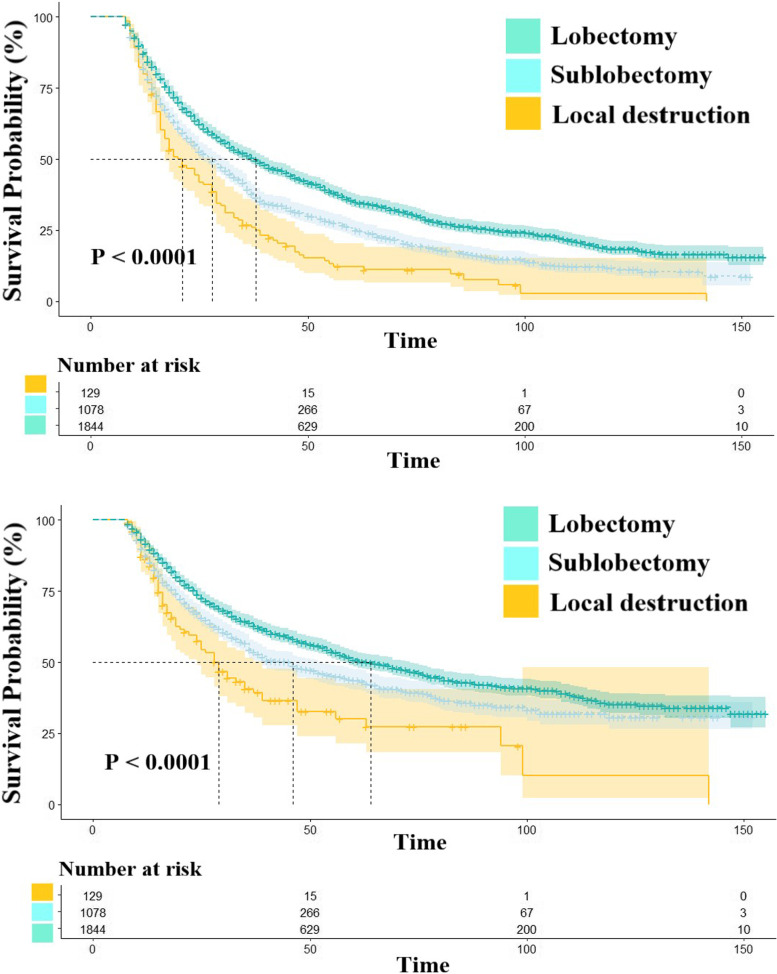
The outcomes of surgery techniques on advanced patients in the beneficial groups. NOTES: Upper: Overall survival; Lower: Cancer-specific survival

Three surgical methods were used to treat patients with advanced NSCLC between 2004–2016. Local destruction, sub-lobectomy, and lobectomy were performed in 129, 1078, and 1844, patients, respectively. Local destruction included laser ablation, microwave ablation, cryoablation, and radiofrequency ablation, whereas sub-lobectomy included segmental and wedge resection. Statistically significant differences were observed between the groups (*P* < 0.001) (Fig. 3). The results from the log-rank test indicated that local destruction had the worst prognosis (MS = 21 months), with 1-, 5-, and 10-year OS rates of 79.8%, 11.2%, and 2.9%, respectively. Moreover, sub-lobectomy with a MS of 28 months and 1-, 5-, and 10-year OS rates of 81.7%, 25.7%, and 11.8%, respectively, had worse long-term outcomes than lobectomy (MS = 38 months; and 1-, 5-, and 10-year OS rates of 87.1%, 35.5%, and 18.6%, respectively).

### Efficacy of mediastinal lymph node dissection in the lobectomy technique

**Fig. 4:**
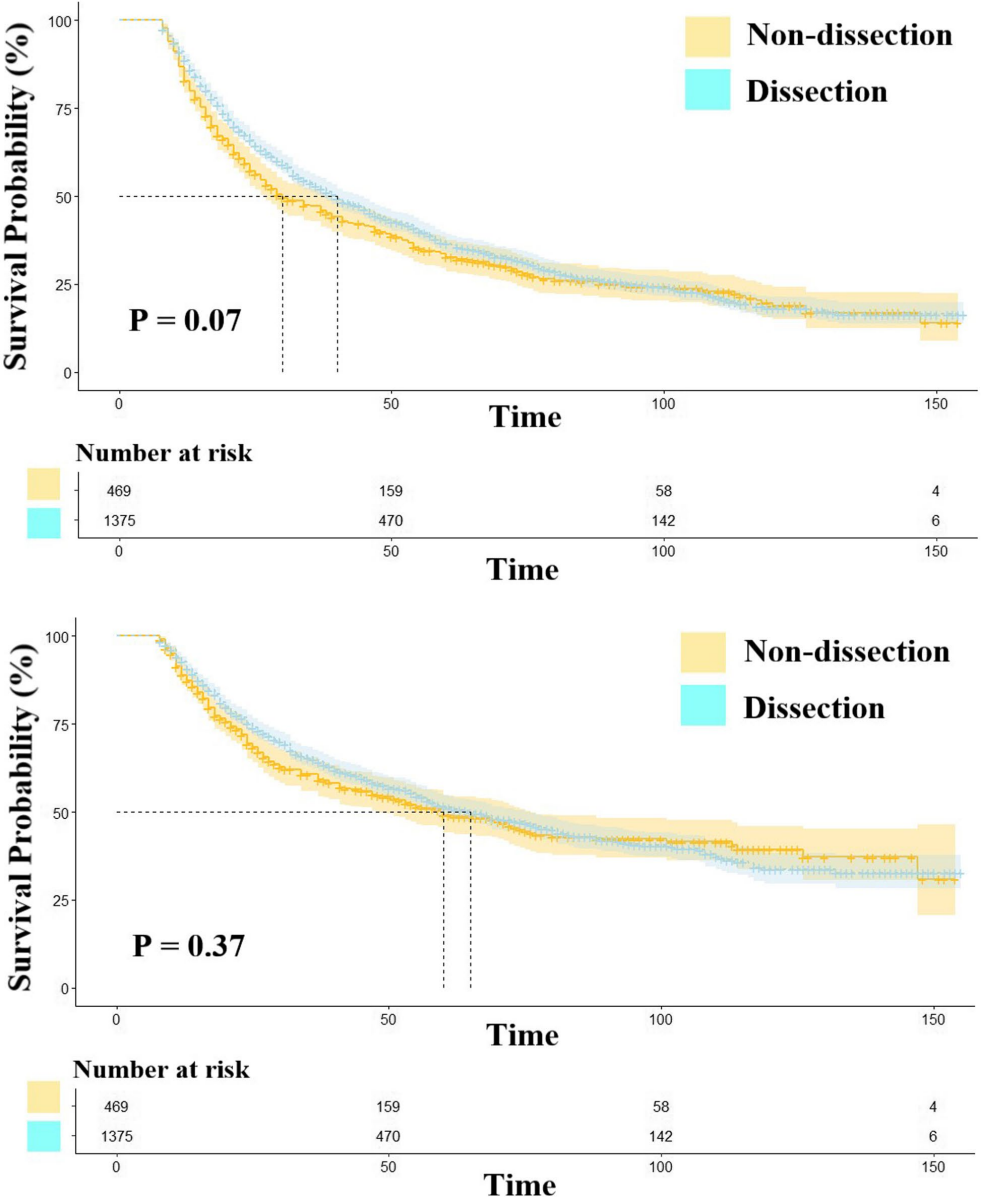
The survival analysis of lymph node resection group and non-resection surgery group before PSM. NOTES: Upper: Overall survival; Lower: Cancer-specific survival

**Fig. 5:**
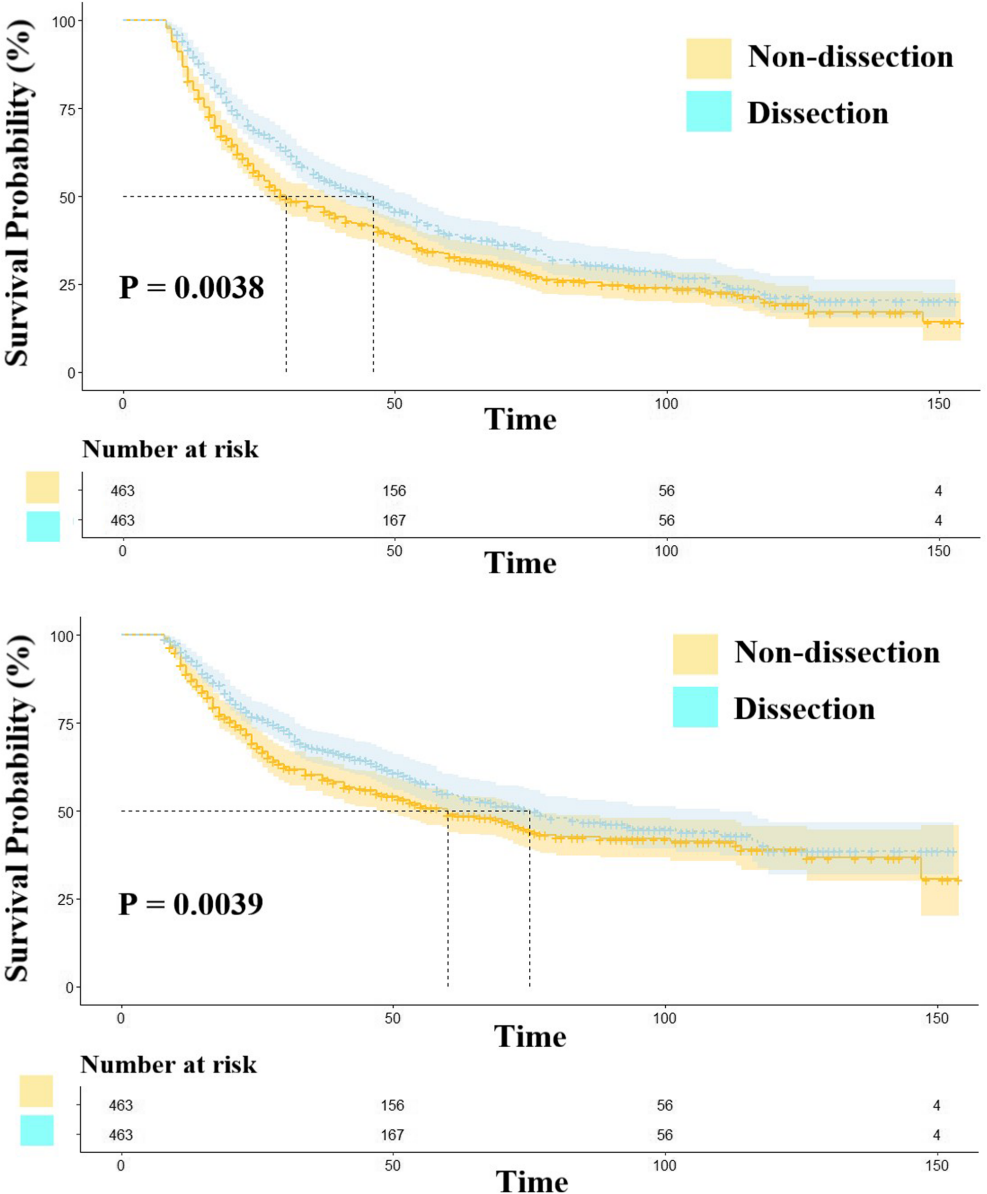
The survival analysis of lymph node resection group and non-resection surgery group after PSM. NOTES: Upper: Overall survival; Lower: Cancer-specific survival

We found no statistically significant differences in OS (*P *= 0.07) and CSS (*P* = 0.37) between the two groups before matching (Fig. 4). Considering the interference of baseline characteristics, we repeated PSM in the lymph node resection and non-resection surgery groups. After PSM, it was clear that lymph node clearing is beneficial for patients with stage IV NSCLC (OS: *P* = 0.0038; CSS: *P* = 0.039) (Fig. 5), as evidenced by the MS and OS times of 30 and 46 months, respectively. However, no significant differences were observed with regard to long-term outcomes, with the non-dissection group showing 1-, 5-, and 10-year OS rates of 82.7%, 32.7%, and 19.0%, respectively, while those in the other group were 88.6%, 36.5%, and 18.4% at 1, 5, and 10 years, respectively.

## Discussion

According to the Global Cancer Statistics of 2020, lung cancer, which accounts for an estimated 1.8 million deaths, remains a major deadly disease, second only to breast cancer [1]. Although previous studies have shown that surgical intervention is beneficial for patients with early cell lung cancer and has a good prognosis, the feasibility of this approach for stage III-IV NSCLC remains controversial [9]. Numerous studies have shown that the prognosis of patients with resectable stage III NSCLC who undergo surgery after neoadjuvant therapy depends on lymph node invasion [10]. Moreover, surgery is generally discouraged in patients with stage IV NSCLC because of their limited survival time [11]. In the present study, we found that palliative surgery improved the prognosis of patients with stage IV NSCLC to some extent, as evidenced by a 2-fold longer MS times in the surgical group than in the non-surgical group. These findings were consistent with those of He et al., [5] who reported a predictive model for identifying optimal patients with stage IV NSCLC for surgery. Results from another study that analysed a SEER dataset, found that patients with stage IV NSCLC had superior OS following thoracic surgery in combination with chemotherapy or chemoradiation. Patients who underwent surgery had longer MS times (15 months) than those in the non-surgery group (8 months) [12].

The 5-year survival rate (22.4%) recorded in the present study was comparable to that reported by Hanagiri et al., [13] who reported long-term follow-up outcomes of 25% in patients with stage IV NSCLC after surgical resection of the primary lesion, as well as aggressive treatment of metastases using radiotherapy, stereotactic radiosurgery (SBRT), or surgery [13]. Additional evidence showed that salvage lung resection of R0 after concurrent neoadjuvant chemoradiotherapy (CRT) was necessary for advanced lung cancer in patients who could tolerate the surgery and was accompanied by a MS time of 24 months. Moreover, their findings further indicated that non-extensive lung resection was sufficient, with a prognostic value comparable to that of extensive surgery [6, 14]. Apart from CRT, targeted therapy is the most common drug-based treatment for advanced patients with positive driver genes, while EGFR mutations are the most frequently targeted genetic factor. The retrospective study conducted by Gong et al., revealed the safety and rationality of palliative surgery after 2–46 months of targeted therapy, with median event-free and postoperative survival rates of 14 and 17 months, respectively [15]. SBRT has also been found to be a selective therapy for metastases [4]. Notably, an acceptable prognosis for salvage surgery was observed following SBRT, with 5-year progression-free survival and OS rates of 15% and 40.6%, respectively [16].

We also compared three types of surgical techniques, namely local destruction (laser ablation, microwave ablation, cryoablation, and radiofrequency ablation), sub-lobectomy (segmental resection and wedge resection), and lobectomy, and found that lobectomy was superior to the others, as evidenced by the highest long-term prognosis. Conversely, the ablation technique was the least effective, although it was still superior to the non-surgery group, which is consistent with previous studies. Ablation has recently emerged as a treatment option for patients with advanced tumours. Indeed, Solomon et al., [17] demonstrated advantages of thermal ablation over surgery for the treatment of lesions < 3 cm (especially < 2 cm) in terms of safety and quality of life. Notably, patients who underwent ablation therapy exhibited longer OS when the lesion was < 3 cm relative to those in the non-surgery group, with 5-year survival rates of 10% and 5% in the ablation and no surgery groups, respectively [18]. Cryoablation is another new alternative to thermal ablation that may enhance treatment responses to immunotherapy in patients with advanced lung cancer (cryoimmunotherapy) [19].

In the present study, although patients who received lobectomy were found to have a good prognosis, ablation therapy was considered a good choice for patients who could not tolerate surgical trauma. However, the lobectomy approach remains controversial given that its benefits in progression-free and OS rates, as well as in enhancing the quality of life, remain unclear for mediastinal lymph node clearing during surgery. The results of the present study support the use of lymph node resection, consistent with the findings of Dr Daniel L and Daigo Kawano. Miller showed that the presence of mediastinal lymph node metastases significantly affected the postoperative 5-year survival rates of patients with distant metastasis [20, 21]. However, further studies are required to validate these findings.

This study has some limitations that warrant discussion. First, as this was a retrospective study, we anticipate some bias compared to that observed with randomised controlled trials. Second, we only extracted clinical information on chemotherapy and radiotherapy but not about other treatment approaches such as targeted therapy and immunotherapy. Third, the efficacy of drug therapy or surgery in patients with distant metastasis is unclear. It is possible that the postoperative prognosis of patients with advanced NSCLC is affected by distant organ types. As surgery on different oligometastatic systems can generate different results [22], further research is needed to ascertain the efficacy of standard treatment in patients with stage IV NSCLC.

## Data Availability

The datasets analyzed during the current study are available from the corresponding author on reasonable request.
